# Effect of Evening Primrose Oil-Based Polyol on the Properties of Rigid Polyurethane–Polyisocyanurate Foams for Thermal Insulation

**DOI:** 10.3390/polym10121334

**Published:** 2018-12-03

**Authors:** Joanna Paciorek-Sadowska, Marcin Borowicz, Bogusław Czupryński, Marek Isbrandt

**Affiliations:** Department of Chemistry and Technology of Polyurethanes, Technical Institute, Faculty of Mathematics, Physics and Technical Science, Kazimierz Wielki University, J. K. Chodkiewicza Street 30, 85-064 Bydgoszcz, Poland; czupr@ukw.edu.pl (B.C.); m.isbrandt@ukw.edu.pl (M.I.)

**Keywords:** evening primrose oil, biopolyol, synthesis, polyurethane–polyisocyanurate foam, foam properties, thermal insulation

## Abstract

The article presents the results of research on the synthesis of a new biopolyol based on evening primrose oil, and its use in the production of rigid polyurethane–polyisocyanurate foams intended for thermal insulation. The obtained biopolyol was subjected to analytical, physicochemical, and spectroscopic tests (Fourier transform infrared (FTIR), ^1^H NMR, ^13^C NMR) to confirm its suitability for the synthesis of polyurethane materials. Then, it was used for the partial replacement of the petrochemical polyol in the polyurethane formulation. Obtained rigid polyurethane–polyisocyanurate foams are characterized by a lower apparent density, brittleness, water absorption, and thermal conductivity coefficient λ. In addition, foams modified by biopolyols had a higher content of closed cells and higher aging resistance. The results of the conducted research showed that the use of the biopolyol based on evening primrose oil may be an alternative to petrochemical polyols. The research presented herein is perfectly consistent with the trends of sustainable development and the philosophy of green chemistry.

## 1. Introduction

Nowadays, the interest of producers in the chemical industry focuses on technologies using environmental-friendly raw materials [1,2]. In the case of polymer materials, particular emphasis has been placed on the impact of raw materials and products on man and the surrounding environment since the beginning of the 1980s [3]. Current trends in the chemical industry are directed at the use of sustainable products, the addition of raw materials from renewable sources, and the solution of the waste problem at the production planning stage. The application of renewable raw materials in the polyurethane (PU) industry is mainly based on synthesis of new polyol compounds. A wide and huge group of renewable raw materials for the synthesis of polyol compounds (biopolyols) for the production of PU materials are vegetable oils. The most commonly used vegetable oils include: soybean, palm, coconut, linseed, castor, rapeseed, and sunflower oils. This group of raw materials includes, above all, the esters of higher unsaturated fatty acids (oleic, linoleic, linolenic) and glycerol. They also contain a few percent of saturated acids (stearic and palmitic) [4]. However, the molecules of oil must be chemically modified to unlock or incorporate reactive hydroxyl groups, which are capable of reacting with isocyanate groups. The methods of chemical modification of double bonds include reactions of: epoxidation, hydrogenation, oxidation, and the halogenation of unsaturated bonds [5,6,7,8,9]. Only such modification ensures obtaining a biopolyol with properties that enable its application in industry [10]. These methods are developed and used in the polyurethane industry, because they are quite cheap. Raw materials (vegetable oils), which are needed in these technologies, are easily available. Some oils, e.g., linseed or mustard oil, have a higher content of unsaturated bonds; therefore the biopolyols that are based on them have a higher hydroxyl number, and the obtained PU foams are more rigid. Others oils, e.g., olive oil, have a lower content of reactive groups, and obtained polyurethane foams can be more flexible [11]. The properties of the obtained polyurethane material can be controlled by selecting appropriate raw materials [12,13,14,15,16,17,18,19].

A large group of polyurethanes is rigid polyurethane foams. These PU materials are a modern and safe method of thermal and acoustic insulation, due to the ease of application. They enable the exact filling of the space, which will be thermally insulated. They are most often used in light construction as insulation materials. They are also used for the production of cores in sandwich panels and for insulating external installations [20]. The functional requirements of PU foams are connected with their most important application (as good and safe thermal insulation materials), while maintaining good mechanical properties and dimensional stability. The quality of the obtained polyurethane material depends on a few factors, for example: the composition of raw materials, their molar ratio, the conditions of synthesis, the additive compounds, and the application method [21]. The huge possibilities of producing polyurethane materials with designed properties are the result of a wide raw material base, especially in the field of polyol raw materials. Ecological aspects also play an important role for the producers of polyurethane foams. The addition of biopolyols obtained from vegetable oils to the base of renewable raw materials is consistent with the direction of the idea of sustainable development for polymeric materials. This is an interesting alternative to petrochemical polyols [22,23,24,25,26].

Earlier research concerned the synthesis of biopolyols based on vegetable oils, in which the ring-opening agent was 2,2’-mercaptodiethanol [24,27,28]. The aim of the research that is presented in this article was obtaining a new biopolyol based on evening primrose oil and diethylene glycol, and using it for the production of rigid polyurethane–polyisocyanurate foams for thermal insulation application.

## 2. Materials and Methods

### 2.1. Materials

Fresh and unrefined evening primrose oil was supplied by Olejarnia Kołodziejewo (Kołodziejewo, Poland) and used for synthesis of the new biopolyol (EPB). The iodine value was 0.658 mol I_2_/100 g of oil, the acid value was 8.720 mg KOH/g, and the content of unsaturated fatty acids was 92.7% of all fatty acids. EPB was obtained in accordance with Polish Patent Application P.422888 [28], wherein the first step used: 99.5% acetic acid (Chempur, Piekary Śląskie, Poland), 30% hydrogen peroxide (Chempur, Poland), and 96% sulfuric acid (Chempur, Poland) as an oxidizing system of double bonds; a second step applied: 99% diethylene glycol p.a. (Chempur, Poland) and 96% sulfuric acid (as above) to open the epoxy rings. Anhydrous magnesium sulfate (Chempur, Poland) was used for deactivation of the catalyst and drying the oil-based polyol.

Polyols: biopolyol based on evening primrose oil, Rokopol RF-551 (sorbitol oxyalkylation product, PCC Rokita S.A., Brzeg Dolny, Poland) and technical polyisocyanate Purocyn B (polymeric 4,4’-diphenylmethane diisocyanate supplied by Purinova, Bydgoszcz, Poland) were used as the main raw materials for the production of rigid polyurethane–polyisocyanurate (RPU/PIR) foams. As an additive, agents in foam premixes were used, which included: 33% solution of 1,4-diazabicyclo[2,2,2]octane (DABCO, Alfa Aesar, Haverhill, MA, USA) in diethylene glycol (Chempur, Poland) as a catalyst of urethane bond formation; 33% solution of anhydrous potassium acetate (Chempur, Poland) in diethylene glycol (as above) as a catalyst of trimerization of NCO groups; Tegostab 8460 (Evonik, Essen, Germany) as a silicone surfactant; Solkane HFC 365/227 (Solvay, Brussels, Belgium) as a blowing agent, and Antiblaze TCMP (Albemarle, Charlotte, NC, USA) as a flame retardant.

### 2.2. Synthesis of Evening Primrose Oil-Based Polyol

**Figure 1:**
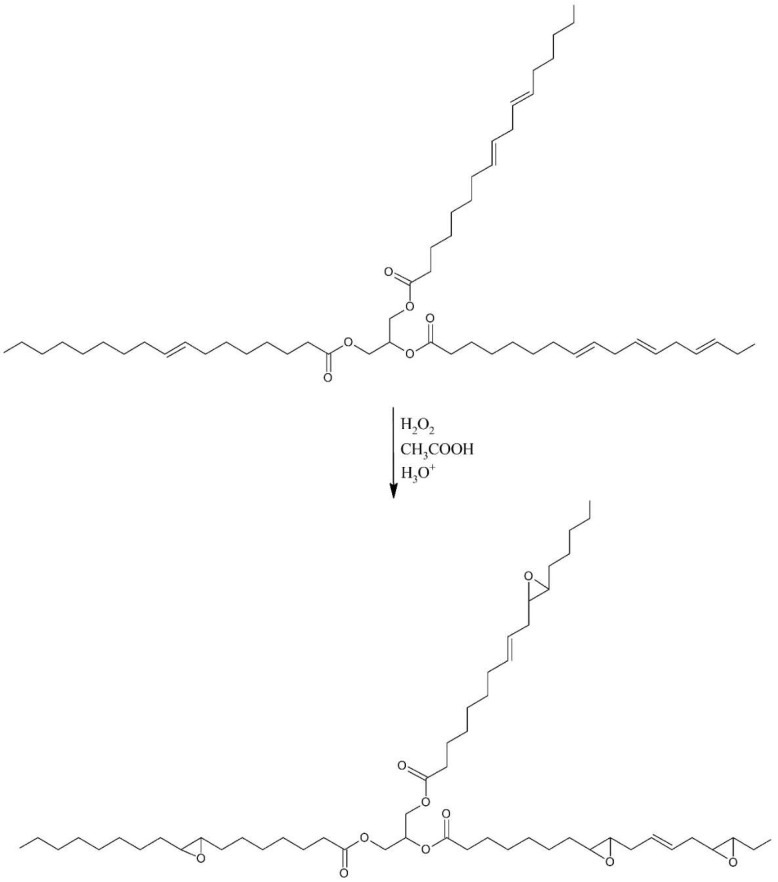
Schematic epoxidation reaction.

The synthesis of a biopolyol based on evening primrose oil (EPB) was carried out in two steps in a glass reactor with a heating jacket, equipped with a reflux condenser, temperature sensor, dropping funnel, and mechanical stirrer. In the first step of synthesis, the evening primrose oil (EPO), acetic acid (AA), and sulfuric acid (SA) were loaded into reactor and heated to 40 °C. Hydrogen peroxide (HP) was gradually added after reaching this temperature. After the addition of HP, the whole mixture was heated to 60 °C. The reaction lasted for three hours. The molar ratio of the reactants calculated with respect to the iodine value of EPO was 1:1:1:0.02 for EPO:AA:HP:SA (mass of the reactants were shown in Table 1). Oil and water phases were separated after reaction. The oil phase was washed with distilled water and dried by solid anhydrous magnesium sulfate. In the first step of the synthesis, epoxidized evening primrose oil (EPEO) was obtained, which was subjected to analytical tests. The reaction scheme was shown in Figure 1.

The efficiency of the epoxidation reaction of evening primrose oil (first step) is defined by Equation (1):

(1) $$E_{1}=\frac{IV_{EPO}-IV_{EPEO}}{IV_{EPO}}\cdot 100\%$$

where: *E*_1_—efficiency of epoxidation reaction, *IV_EPO_*—iodine value of evening primrose oil, and *IV_EPEO_*—iodine value of epoxidized oil.

**Figure 2:**
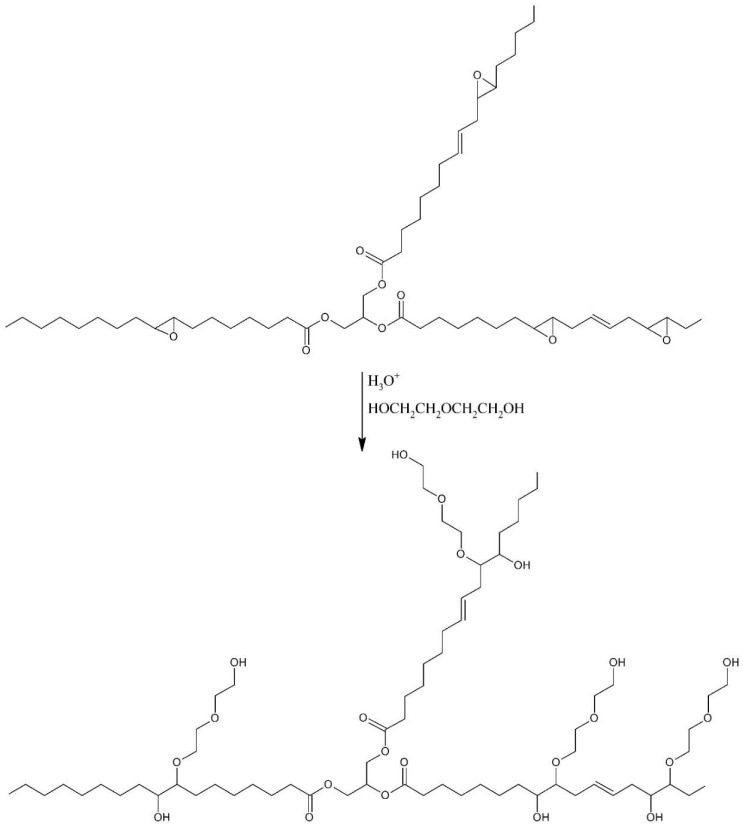
Schematic ring-opening reaction.

In the next step, epoxidized evening primrose oil (EPEO), diethylene glycol (GDE) and the reaction catalyst (SA) were loaded into a glass reactor with a heating jacket, which was equipped with a reflux condenser, temperature sensor, and mechanical stirrer. The molar ratio of reactants calculated with respect to the epoxy value of EPEO was 1:1:0.01 for EPEO:GDE:SA (Table 1). Afterwards, the whole mixture was heated to 100 °C. Reaction was carried out for four hours until the opening of all of the epoxide rings by diethylene glycol. After the synthesis, the obtained biopolyol (EPB) was neutralized by solid anhydrous magnesium sulfate and distilled under vacuum to reduce the water content. The second reaction scheme is shown in Figure 2.

The efficiency of the opening reaction of epoxide rings (second step) was defined by Equation (2):

(2) $$E_{2}=\frac{EV_{EPEO}-EV_{EPB}}{EV_{EPEO}}\cdot 100\%$$

where: *E*_2_—efficiency of epoxide rings’ opening reaction, *EV_EPEO_*—epoxy value of epoxidized evening primrose oil, *EV_EPB_*—epoxy value of biopolyol.

### 2.3. Synthesis of RPU/PIR Foams

The formulation of RPU/PIR foam premixes with evening primrose oil-based polyol (EPB) required experimental investigations to determine the optimal composition of additive agents (catalysts, surfactant, flame retardant, and blowing agent). The hydroxyl number was the basis for determining the amount of polyol raw materials in the formulation. These values enabled calculating the mass equivalents (*R*) of the hydroxyl group in polyols. The sum of mass equivalents of petrochemical polyol and biopolyol was always one. The addition of isocyanate raw material was selected in consideration of the mass equivalent of NCO groups. The ratio of NCO to OH groups in the reaction mixture for RPU/PIR foams was 3:1. An excess of isocyanate raw material was necessary for a reaction between NCO and OH groups (to produce a urethane bond) and the trimerization of three NCO groups (to produce an isocyanurate ring). 

The content of additive agents was calculated in relation to the sum of masses of polyols and polyisocyanate (in weight percentages): silicone surfactant (1.7 wt.%), urethane bond catalyst (1 wt.%), isocyanate trimerization catalyst (2.5 wt.%), flame retardant (17 wt.%), and physical blowing agent (12 wt.%). The formulation of RPU/PIR foams was shown in Table 2.

RPU/PIR foams were obtained at a laboratory scale by using the one-step method, from the two-component system. Component A was obtained as a result of mixing appropriate amounts of the polyol, biopolyol, silicone surfactant, two catalysts, flame retardant, and blowing agent in a polypropylene cup. Component B was a technical polyisocyanate raw material. Components A and B were mixed together and stirred for 10 s with a mechanical stirrer (1800 rpm) in a suitable mass ratio. After that, the mixture was poured into a cuboidal mould with a movable bottom with internal dimensions of 25 cm × 25 cm × 30 cm, where the growth of foam proceeded freely. Five types of foams were obtained in this research: EPB2.0—foam without biopolyol, and EPB2.1–2.4—foams with increasing biopolyol content based on evening primrose oil by the partial replacement of petrochemical polyol. The synthesis of RPU/PIR foams was thrice repeated. Obtained polyurethane materials were thermostated for six hours at 120 °C in a laboratory dryer with forced circulation, after removal from the mold.

### 2.4. Methods

#### 2.4.1. Analysis of Synthesis Process and Product

Analytical and physicochemical tests were performed on the evening primrose oil, epoxidized oil, and biopolyol. It was aimed to determine its suitability for the synthesis of RPU/PIR foams.

The hydroxyl number (HN) was determined in accordance with the industrial standard of Purinova Ltd. No. WT/06/07/PURINOVA, by an acylation method with acetic anhydride in *N*,*N*′-dimethylformamide as a medium. An excess of acetic anhydride after hydrolysis and obtained acetic acid were titrated by using a standard potassium hydroxide solution and phenolphthalein as an indicator.

The acid value (AV) was determined in accordance with PN-EN ISO 660:2010. The analysis was performed by titration of the sample dissolved in a mixture of ethyl ether-ethyl alcohol (1:1) by using the standard solution of potassium hydroxide in ethyl alcohol and phenolphthalein as an indicator.

The iodine value (IV) was determined in accordance with PN-EN ISO 3961:2018-09 by the reaction of unsaturated bonds with iodine monochloride (Wijs solution) and the titration of iodine excess.

The epoxy value (EV) was determined in accordance with PN-EN ISO 3001:2002 by the reaction of epoxy rings with tetraethylammonium bromide and the titration of bromide excess.

The viscosity of the evening primrose oil, epoxidized oil, and biopolyol was determined by using a Fungilab digital rheometer at 20 °C (293 K). The measurements were carried out using a standard spindle (DIN-87) working with the bushing (ULA-DIN-87). A constant temperature was maintained through the thermostat connected to the water jacket of the sleeve.

The densities of the evening primrose oil, epoxidized oil, and biopolyol were measured at 25 °C (298 K) in an adiabatic pycnometer in accordance with ISO 758:1976.

The water content was determined by the Karl Fischer method using a non-pyridine reagent under the trade name Titraqual in accordance with PN-81/C-04959.

The pH value was measured using a Hanna Instruments microprocessor laboratory pH-meter (ORP/ISO/°C) with an RS 22 C connector.

The average molecular weight (*M_w_*) of the biopolyol based on evening primrose oil was determined by gel permeation chromatography (GPC) by using a Knauer chromatograph. The apparatus was equipped with thermostated columns and a refractometer detector. The measurements were made on the basis of calibration, by using polystyrene standards in the range of *M_w_* from 162 g/mol to 25,500 g/mol. Also on the basis of HN and average *M_w_*, the functionality (*f*) of biopolyol was calculated by Equation (3):

(3) $$f=\frac{M_{W}\cdot HN}{56100}$$

For confirmation, the course of the synthesis and chemical structure of the obtained oil-based polyol, the evening primrose oil, epoxidized oil, and biopolyol were tested in Fourier transform infrared (FTIR) spectroscopy using Brücker Vector spectrophotometer by KBr technique in the 400 to 4000 cm^−1^ range and in nuclear magnetic resonance spectroscopy ^1^H NMR and ^13^C NMR using a Brücker NMR Ascend III spectrometer with a frequency of 400 MHz, in deuterated chloroform, as a solvent.

#### 2.4.2. Foaming Process

The foaming process was analyzed in accordance with ASTM D7487 13e—Standard Practice for Polyurethane Raw Materials: Polyurethane Foam Cup Test [29]. During obtaining RPU/PIR foams, cream, free rise, string gel, and tack free times were measured by an electronic stopwatch.

#### 2.4.3. Properties of RPU/PIR Foams

The obtained rigid polyurethane–polyisocyanurate foams were tested for application as thermal insulation materials.

The apparent density of foams (the ratio of foam weight to its geometrical volume) was determined for cube-shaped samples with a side length of 50 mm in accordance with ISO 845:2006.

Compressive strength was determined by using the universal testing machine Instron 5544 in accordance with ISO 844:2014. The maximum force inducing a 10% relative strain was determined (decreasing of the foam height in relation to the initial height, according to the direction of foam growth).

The brittleness of the foams was determined in accordance with ASTM C-421-61, as a percentage mass loss of 12 cubic foam samples with a side length of 25 mm. Tests were conducted in a standard cuboidal box made of oak wood with dimensions of 190 mm × 197 mm × 197 mm, rotating around the axis at a speed of 60 rpm. The filling of the box during the measurement were 24 normalized oak cubes with dimensions of 20 mm × 20 mm × 20 mm. The brittleness (B) of obtained foams was calculated from Equation (4):

(4) $$B=\frac{m_{1}-m_{2}}{m_{1}}\cdot 100\%$$

where: *m*_1_—mass of the sample before test (g), and *m*_2_—mass of the sample after test (g).

The flammability of RPU/PIR foams was determined by using three flammability tests: Bütler’s combustion test (vertical test) in accordance with ASTM D3014-04; the horizontal combustion test in accordance with PN-EN ISO 3582:2002/A1:2008, and a limited oxygen index Bütler’s combustion test, which consisted of burning a foam sample with the dimensions of 150 mm × 20 mm × 20 mm in a vertical column (chimney) with dimensions of 300 mm × 57 mm × 54 mm. Combustion residue (CR) was calculated from Equation (5):

(5) $$CR=\;\frac{m_{b}}{m_{a}}\cdot 100\%$$

where: *m_a_*—mass of the sample before the burning test (g), and *m_b_*—mass of the sample after the burning test (g). The horizontal burning test consisted of determining the susceptibility of foam to flame. The result of this test was an evaluation of material flammability (non-flammable, flammable, or self-extinguishing). A limited oxygen index (LOI) was measured by using Concept Equipment apparatus in accordance with ISO 4589. The percentage limited concentration of oxygen was determined in the mixture consisting of oxygen and nitrogen, which was sufficient to sustain the burning of the sample. *LOI* was calculated according to the Equation (6).

(6) $$LOI=\frac{\left[O_{2}\right]}{\left[O_{2}\right]+\left[N_{2}\right]}\cdot 100\%$$

Absorbability (*A*) and water absorption (*WA*) were determined in accordance with ISO 2896:2001, which was measured after immersion in distilled water for 24 h. Values of these parameters were calculated from Equations (7) and (8):

(7) $$A=\frac{m_{A}-m_{D}}{m_{D}}\cdot 100\%$$

where: *m_A_*—mass of the sample after immersion in distilled water (g), and *m_D_*—mass of the dry sample (g).

(8) $$WA=\frac{m_{WA}-m_{D}}{m_{D}}\cdot 100\%$$

where: *m_WA_*—mass of the sample after surface drying (g).

Aging resistance of the foams was carried out in thermostating process of cubic samples with a side length of 50 mm in 48 h at a temperature of 120 °C. The result of this test included a change of linear dimensions (Δ*l*), change of geometrical volume (Δ*V*), and mass loss (Δ*m*). The values of these parameters were calculated in accordance with ISO 1923: 1981 and PN-EN ISO 4590: 2016-11. The formulas for the calculations of Δ*l*, Δ*V*, Δ*m* are shown in Equations (9)–(11).

(9) $$\Delta l=\frac{l-l_{0}}{l_{0}}\cdot 100\%$$

where: *l*_0_—length of the sample before thermostating (according to the direction of foam rise) (mm), and *l*—length of the sample after thermostating (according to the direction of foam rise) (mm).

(10) $$\Delta V=\frac{V-V_{0}}{V_{0}}\cdot 100\%$$

where: *V*_0_—geometrical volume of the sample before thermostating (mm^3^), and *V*—geometrical volume of the sample after thermostating (mm^3^).

(11) $$\Delta m=\frac{m_{0}-m}{m_{0}}\cdot 100\%$$

where: *m*_0_—mass of the sample before thermostating (g), and *m*—mass of the sample after thermostating (g).

The content of closed cells was determined in accordance with PN-EN ISO 4590:2016-11 by using the helium pycnometer AccuPyc 1340 with the FoamPyc option from Micrometrics. This software calculated the content of closed cells based on the measurement of pressure changes in the test chamber.

Thermal conductivity of the foams was determined based on the determination of the thermal conductivity coefficient λ in accordance with ISO 8301. Tests were carried out with the FOX 200 apparatus from LaserComp, in the measurement range of λ equal to 20–100 mW/(m·K). Measurements were performed in the series at intervals of 0.5 s and at an average measuring temperature of 10 °C (temperature of hot plate—20 °C, temperature of cold plate—0 °C).

The foam structure was analyzed by scanning electron microscope (SEM) HITACHI SU8010 (Hitachi High-Technologies Co., Tokyo, Japan). The studies were performed at the accelerating voltage of 30 kV, with the working distance of 10 mm and magnification of 150×. The statistical analysis of cell sizes, wall thickness, and content of cell per area unit was carried out on the basis of obtained micrographs by using ImageJ software (LOCI, Madison, WI, USA).

## 3. Results and Discussion

### 3.1. Synthesis of Biopolyol

**Figure 3:**
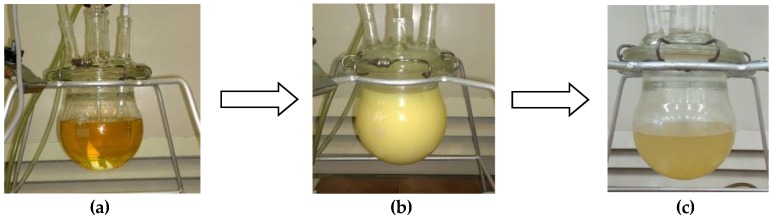
Visual presentation of appearance changes during synthesis (**a**) EPO, (**b**) EPEO, and (**c**) EPB.

A new biopolyol based on evening primrose oil was obtained as the result of a two-step synthesis, involving the epoxidation of double bonds and the opening of obtained epoxide rings with diethylene glycol. The use of this oil was due to the high content of unsaturated fatty acids (double bonds). Their high content promotes the production of biopolyols with high hydroxyl numbers, which is desirable in the synthesis of RPU/PIR foams [29]. The changes of raw materials during the synthesis were presented at Figure 3.

The course of the synthesis was controlled by measuring the appropriate analytical parameters, i.e., measuring iodine value and epoxide value in the first step; and measuring the epoxide value and hydroxyl number in the second step. Evening primrose oil, epoxidized oil, and biopolyol were also subjected to basic physicochemical tests. The results of these tests are shown in Table 3.

A decrease of iodine value and increase of epoxy value was observed as a result of the epoxidation reaction. It was caused respectively by a decrease in the amount of double bonds in the fatty acid residues and an increase in the epoxide rings amount formed in their place. Also, a clear increase in the hydroxyl value was noted. This is due to the attachment of the diethylene glycol molecules to the chain of fatty acids, as a result of the rings’ opening reaction. However, the obtained hydroxyl number was lower than theoretical calculations (tHN = 267.479 mg KOH/g). This is due to the side reaction of biopolyol molecules’ oligomerization and the esterification of free fatty acids in unrefined oil (which led to a significant decrease in the acid value) [30]. It is noteworthy that the obtained values of biopolyol technological parameters are in the range of industrial standards.

Analysis of evening primrose oil, epoxidized oil, and biopolyol was also carried out in spectroscopic tests (FT-IR, ^1^H NMR, and ^13^C NMR).

**Figure 4:**
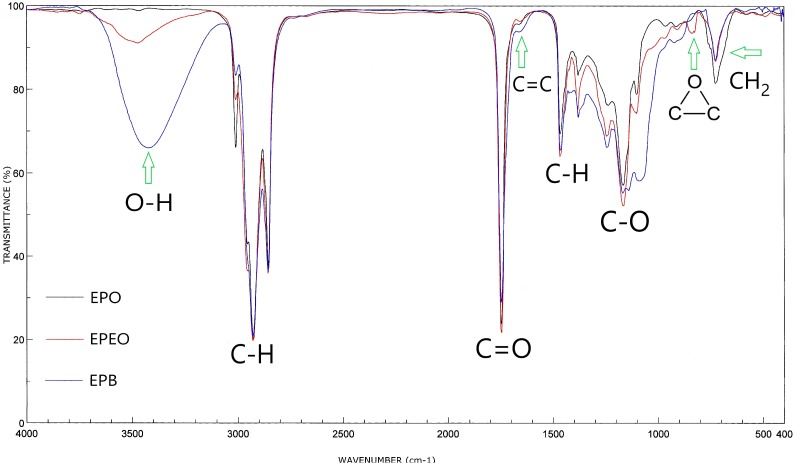
Fourier transform infrared (FTIR) spectra of EPO, EPEO, and EPB.

Infrared spectroscopy analysis of EPO, EPEO, and EPB (Figure 4) showed that these compounds contained characteristic bonds for the structure of fatty acid glycerides. Bands at: 1740 cm^−1^ (stretching) belonged to the C=O groups; 1241, 1163, and 1099 cm^−1^ (stretching) belonged to the C–O bonds of the ester group; 3010 cm^−1^ (stretching) belonged to the C–H bonds in the olefin group; 2950 cm^−1^ (stretching) and 1465 cm^−1^ (deformational) belonged to the C–H bonds in the –CH_2_- groups; 2925 cm^−1^ (stretching) and 1380 cm^−1^ (deformational) belonged to the C–H bond of the –CH_3_ groups. Furthermore, the stretching vibration of the C=C bond from the olefin group and the pendulum vibrations of the CH_2_ groups were observed respectively at 1650 and 725 cm^−1^. In the EPEO spectrum, the presence of doublet bands coming from epoxy groups at 900 and 850 cm^−1^ was noted. These bands did not occur in the spectra of EPO and EPB. A low-intensity band at 3450 cm^−1^ was also noted. This band belonged to the stretching vibrations of the hydroxyl groups. However, its low intensity suggests that it was a residue of water after purification. The FTIR spectrum of the biopolyol showed a high intensity of band at 3450 cm^−1^. This suggests the presence of a large number of O–H bonds. In addition, the band of the C–H bond from olefin groups (3010 cm^−1^) was significantly reduced [31].

**Figure 5:**
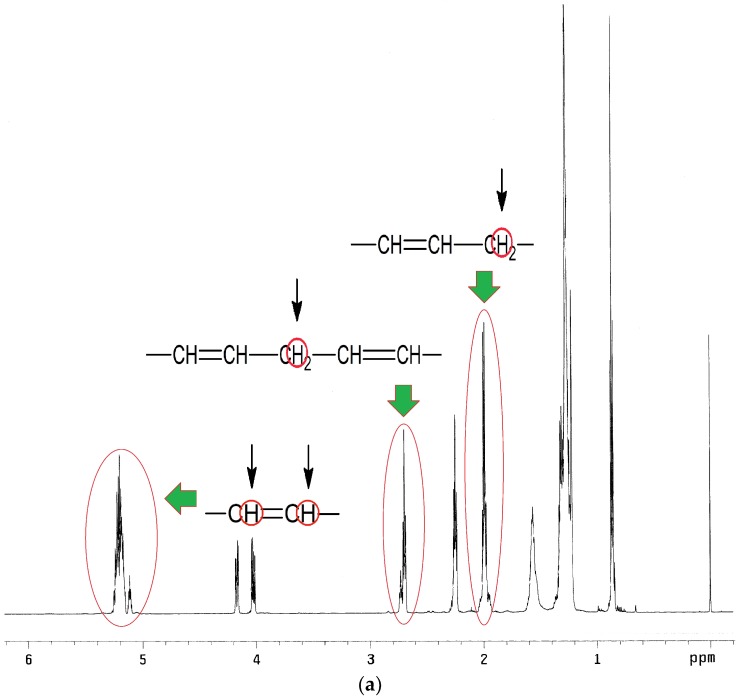

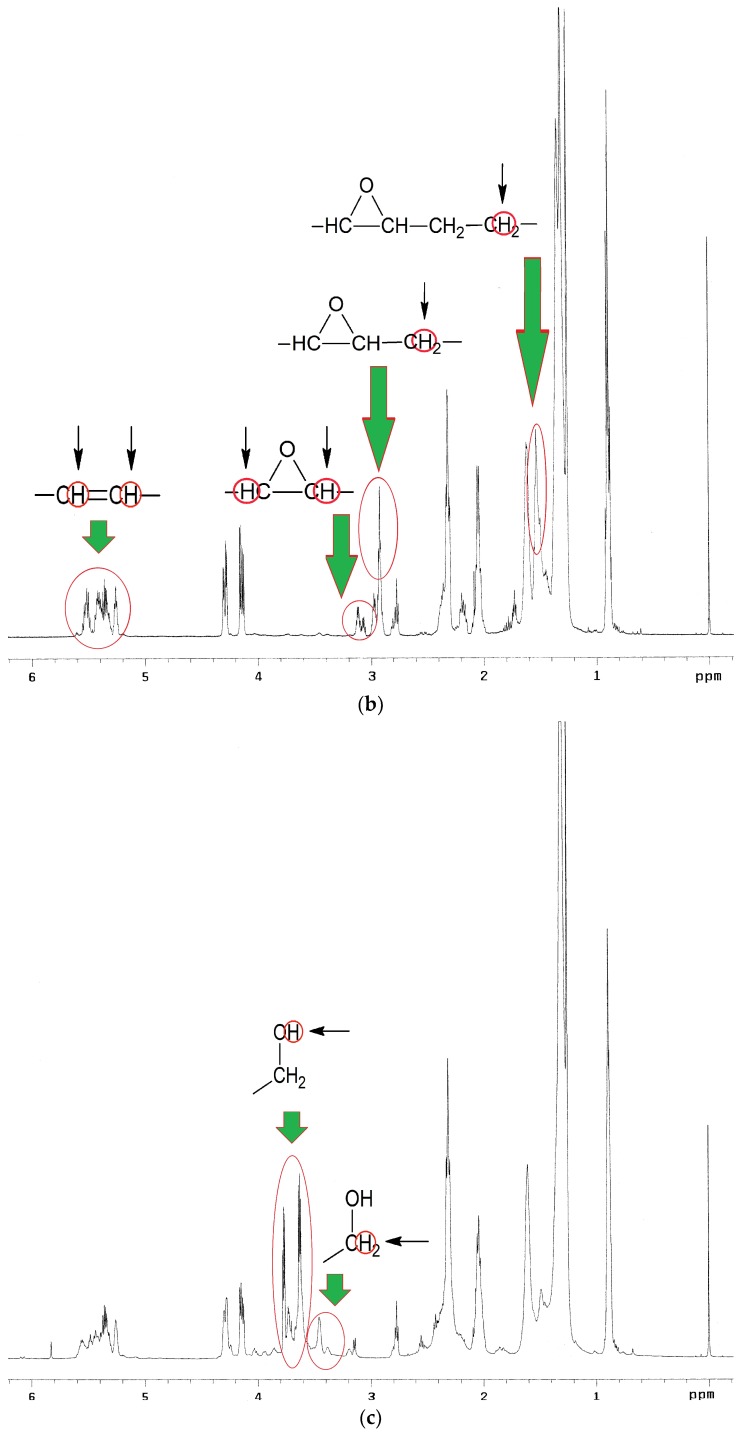
^1^H NMR spectra of (**a**) EPO, (**b**) EPEO, and (**c**) EPB.

^1^H NMR spectra analysis of EPO, EPEO, and EPB (Figure 5a–c) showed characteristic chemical shifts for: 5.35 ppm protons of the olefin groups of fatty acids –C**H**=C**H**–; 5.25 ppm methane protons of glyceryl –CH_2_–C**H**–CH_2_–; 4.12–4.28 ppm methylene protons of glyceryl –C**H**_2_–CH–C**H**_2_–; 2.70–2.80 ppm protons of bis-allyl methylene groups –CH=CH–C**H**_2_–CH=CH–; 2.29–2.34 ppm protons of the α-CH_2_ group to the carbonyl group –C**H**_2_-CO–; 1.95–2.15 ppm protons of α-CH_2_ groups to the olefin group –CH_2_–C**H**_2_–CH=CH–; 1.50–1.60 ppm protons of the β-CH_2_ group to the carbonyl group –C**H**_2_–CH_2_–CO–; 1.25–1.40 ppm protons of C**H**_2_ groups in the fatty acid chain; and 0.86–0.88 ppm protons of ending –C**H**_3_ groups. A characteristic chemical shift for the epoxy groups was only observed on the EPEO spectrum: 3.1–3.2 ppm protons of epoxy group –C**H**–(O)–C**H**–; 2.85–2.95 ppm protons of α-CH_2_ groups to the epoxy group -–CH–(O)–CH–C**H**_2_– and 1.55 ppm protons of α-CH_2_ groups to the epoxy group –CH–(O)–CH–CH_2_–C**H**_2_–. Additional chemical shifts were only noticed in the EPB spectrum: 3.70–3.80 ppm protons of hydroxyl groups at the end of the chain –O**H**; and 3.42–3.50 ppm protons of α-CH_2_ groups to the hydroxyl group –C**H**_2_–OH [32].

**Figure 6:**
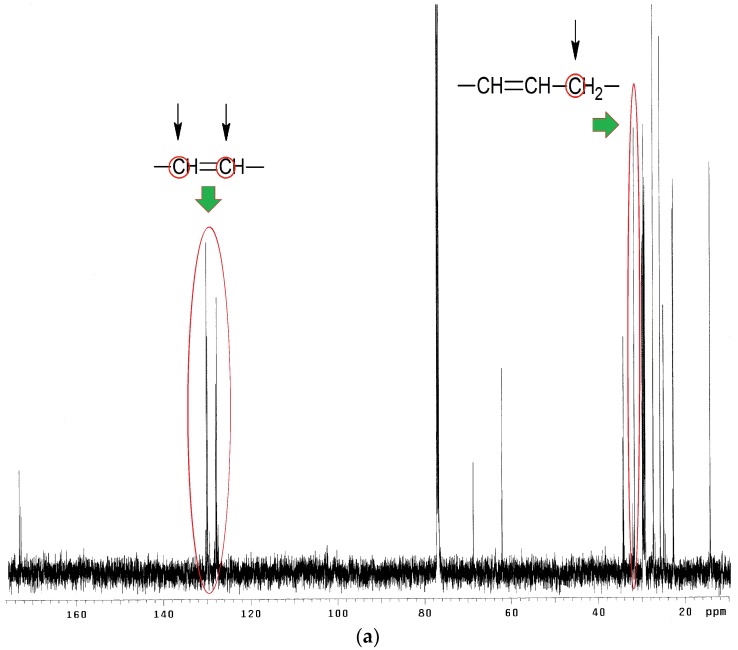

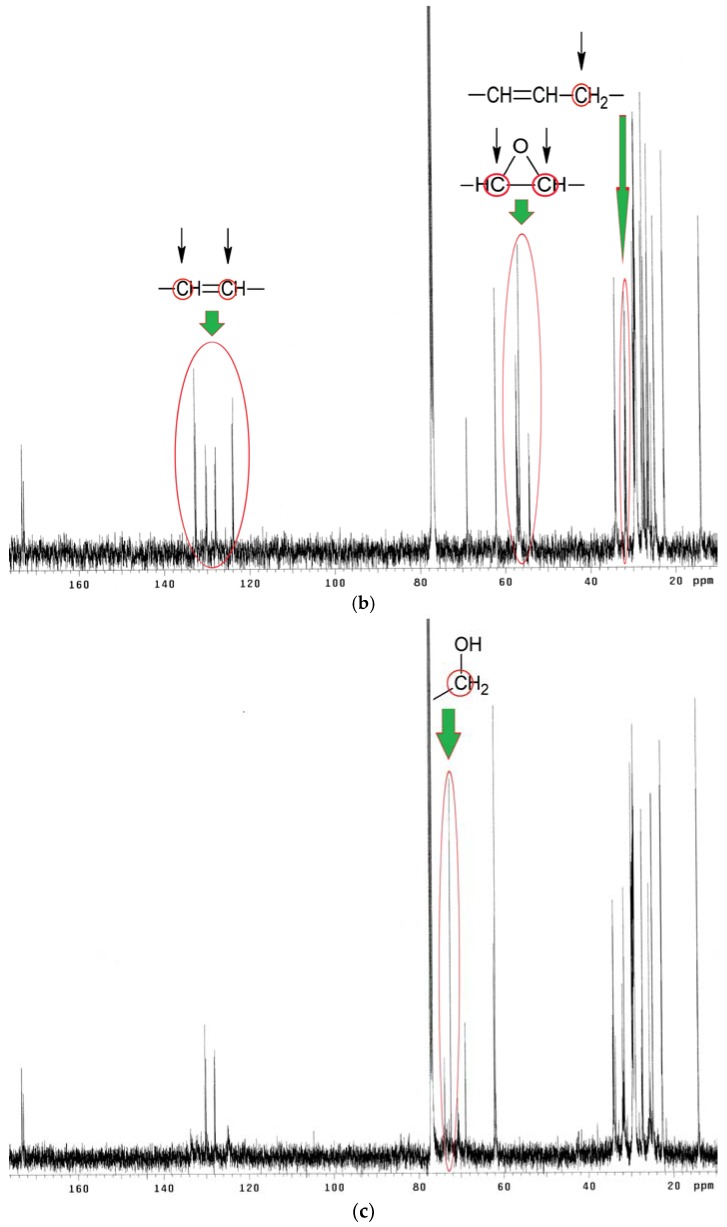
^13^C NMR spectra of (**a**) EPO, (**b**) EPEO, and (**c**) EPB.

^13^C NMR spectra analysis of EPO, EPEO, and EPB (Figure 6a–c) showed characteristic chemical shifts for: 172.80–173.25 ppm carbons of carbonyl groups >**C**=O; 125.10–132.30 ppm carbons of olefin group of fatty acids –**C**H=**C**H–; 68.90 ppm methane carbons of glyceryl –CH_2_–**C**H–CH_2_–; 62.10 ppm methylene carbons of glyceryl –**C**H_2_–CH–**C**H_2_–; 33.00 ppm carbons of α-CH_2_ groups to olefin group –CH=CH–**C**H_2_–; 31.90 ppm carbons of α-CH_2_ groups to the carbonyl group –CH_2_–OOC–**C**H_2_–; 27.20–29.80 ppm carbons of CH_2_ groups in the fatty acid chain; 22.70 ppm carbons of penultimate groups –**C**H_2_–CH_3_; 14.30 ppm carbons of ending groups –**C**H_3_. Characteristic chemical shifts for epoxy structures were noted in the EPEO spectrum: 55.21–57.35 ppm carbons of epoxy group –**C**H–(O)–**C**H–; 34.00 ppm carbons of α-CH_2_ groups to the epoxy group –**C**H_2_–CH–(O)–CH–. Furthermore, the chemical shift of carbons of α-CH_2_ groups to a hydroxyl group –**C**H_2_–OH in 73.86 ppm was only noticed in Figure 6c [33].

The spectroscopic tests confirmed the assumed structure of epoxidized oil and a biopolyol based on evening primrose oil presented in Figure 1 and Figure 2.

### 3.2. Foaming Process

The synthesis of RPU/PIR foams was monitored by measuring the appropriate technological times with an electronic stopwatch. The result of this measurement are shown in Table 4.

The research showed that the addition of biopolyol based on evening primrose oil did slightly change the technological times. It also meant that an increase in the amount of EPB biopolyol with a higher functionality (*f* = 5.3) than the petrochemical Rokopol RF-551 (*f* = 4.5) does not significant affect the synthesis of RPU/PIR foams.

### 3.3. Properties of RPU/PIR Foams

The obtained rigid foams with a different content of polyol based on evening primrose oil were tested for application as thermal insulation materials. The foam obtained without biopolyol was used as a reference.

#### 3.3.1. Physicomechanical Properties of RPU/PIR Foams

One of the most important parameters of RPU/PIR foams for thermal insulation application is apparent density. This parameter indirectly influences the other properties of these materials, such as for example, compressive strength in a parallel direction to foam growth and brittleness. In this case, the increase in the content of a plant-based polyol resulted in a decrease of the apparent density from 45.12 kg/m^3^ for the reference foam to 40.03 kg/m^3^ for the foam with the highest biopolyol content. The main reason for this decreasing was the addition of a component containing long, linear chains (fatty acid residues), which caused a decrease in the packing degree of polyurethane macromolecules (low or non-cross-linking potential).

**Figure 7:**
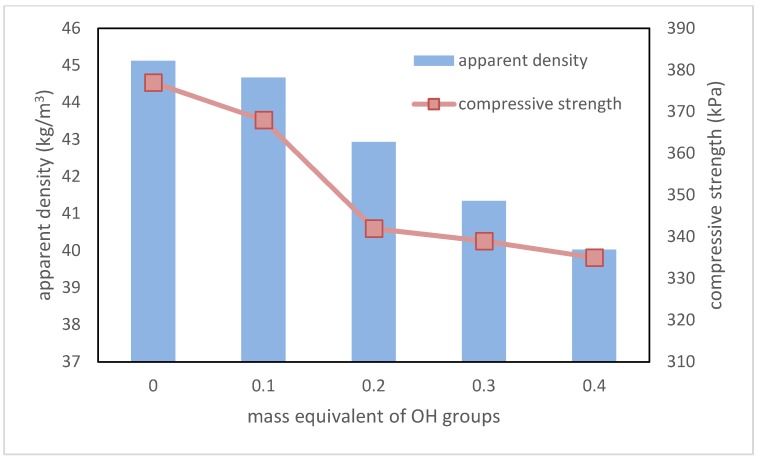
Dependence between EPB content, apparent density, and compressive strength in parallel direction to foam growth.

It also had an influence on the compressive strength of RPU/PIR foams. With the increase in the amount of flexible segments, this parameter decreased from 377 kPa for foam without biopolyol to 335 kPa for foam with the highest content of biopolyol. Dependence between the biopolyol content and the apparent density and compressive strength in the parallel direction to foam growth is shown in the Figure 7.

Despite the decrease in the compressive strength of RPU/PIR foams, the value of this parameter is at a satisfactory level, which for this type of polyurethane materials is above 280 kPa.

Increased flexibility, which was resulting from the addition of linear polyol chains, influenced the decrease in brittleness of obtained RPU/PIR foams. When the evening primrose oil-based polyol was used, a significant decrease of this parameter was noted (from 40.17% for the foam with biopolyol to 22.41% for foam with 0.4 R of biopolyol).

**Figure 8:**
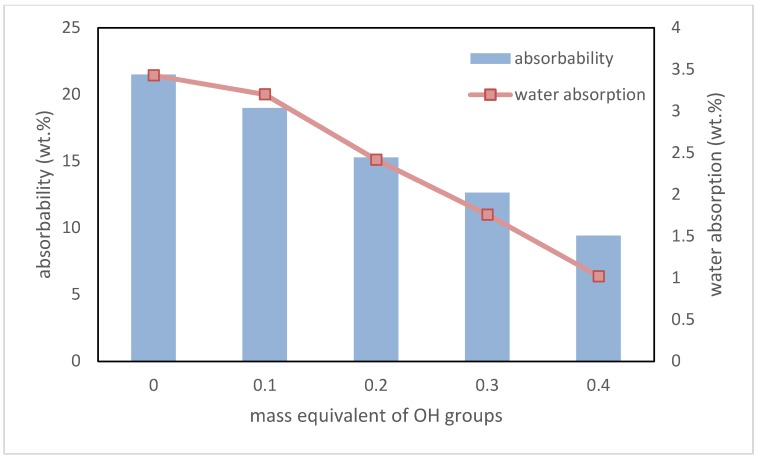
Dependence between EPB content, absorbability, and water absorption.

Another important parameter of foams, which are used in thermal insulation applications, are absorbability and water absorption. The former relates to the percentage amount of water in the material after removal from immersion. The second one is the percentage amount of water that stayed inside the foams. In both cases, a decrease in these values was noted. Interdependence between the content of biopolyol based on evening primrose oil, absorbability, and water absorption is shown in Figure 8.

These decreases were mainly caused by the addition of hydrophobic groups derived from plant oil into the macromolecule of polyurethane. Reducing absorbability and water absorption is very beneficial when these materials are using as thermal insulation. A lack of ability for water to accumulate in foams prevents a multiplication of mold and other microorganisms in the rooms where they are used [34].

#### 3.3.2. Flammability of RPU/PIR Foams

High flammability is a problem for most polymeric materials. In the case of materials used as thermal insulation in civil engineering, this is even more important, because the health and life of people during a fire depend on the flammability of these materials. Therefore, it is indispensable to use the flame-retardant compounds. RPU/PIR foams have an isocyanurate ring in their structure, which affects a partially reduction of flammability in comparison with classic PU foams. Flammability tests of RPU/PIR foams, including Bütler’s combustion test, the horizontal combustion test, and the limited oxygen index showed that the new biopolyol did not affect the flammability of these materials (Table 5) [35].

The presented results of the flammability tests showed a slight decrease in the combustion residue and limited oxygen index values. However, these changes were in the range of measurement error. So, it could be assumed that the use of a biopolyol based on evening primrose oil did not affect the flammability of RPU/PIR foams. This is important information from the application point of view, because the use of an oleochemical raw material did not increase the flammability of the foams. However, the evening primrose oil itself is flammable.

#### 3.3.3. Structure of RPU/PIR Foams

**Figure 9:**
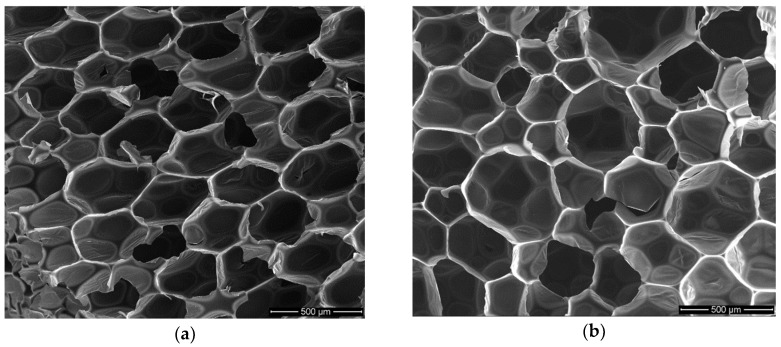
SEM micrographs of (**a**) EPB2.0—reference foam; (**b**) EPB2.4—foam with the highest content of biopolyol.

Foam structure was analyzed with regard to EPB2.0 foam without biopolyol (Figure 9a) and foam with the highest content of biopolyol (Figure 9b).

It was ascertained based on analysis of SEM micrographs that the increase in biopolyol content caused a slight increase in the cell size and its wall thickness. The results of this analysis and comparison of EPB2.0 and EPB2.4 micrographs i.e., average cell size, average cell wall thickness, shape of cells, and average amount of cell per area unit are shown in Table 6.

Slight changes in the structure of foams were caused by the presence of long linear molecules from fatty acid residues in biopolyol. The higher elasticity of the polyol segments and the lower cross-linking of the biopolyol allowed the greater migration of an easily volatile blowing agent. Consequently, the RPU/PIR foams with biopolyol based on evening primrose oil had slightly larger cell diameters and thicker cell walls. It affected inter alia the decrease of apparent density, compressive strength, and brittleness.

#### 3.3.4. Thermal Insulation Properties

**Figure 10:**
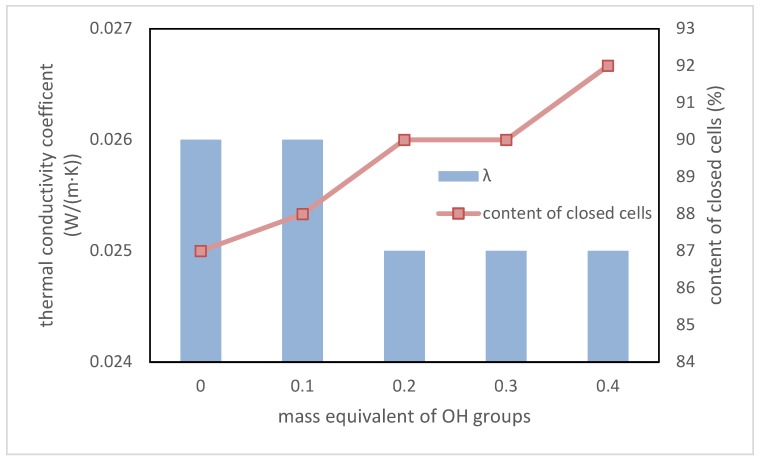
Dependence between EPB content, thermal conductivity coefficient, and content of closed cells.

The most important parameter of the polyurethane materials that are intended for application as thermal insulation is the thermal conductivity coefficient λ. If its value is lower, then the material is a better heat insulator [36]. The content of closed cells in foams is as important as the λ parameter. An increase in the value of this parameter causes a decrease of the λ value. Dependence between the λ value, the content of closed cells, and the content of a biopolyol based on evening primrose oil is presented in Figure 10.

The presented test results showed that the addition of biopolyol improved the thermal insulation properties of RPU/PIR foams. It is true that the change is slight. However, it confirmed that the biopolyol based on evening primrose oil may be an interesting alternative for petrochemical polyols, because the materials based on it have properties that are not worse than commercial foams.

An important parameter of RPU/PIR foams from the thermal insulation point of view is also aging resistance. The results of accelerated aging tests are shown in Table 7.

The addition of vegetable oil-based segments has improved aging resistance. This is due to the fact that the fatty acid glyceride molecule has a higher resistance to external factors, including high temperature, than the polyether polyol. Ether groups contained in Rokopol RF-551 are more susceptible to degradation than ester and ether groups contained in a biopolyol based on evening primrose oil. The reason for this is a greater content of ether bonds in petrochemical polyol (average, one ether bond for every two carbons) than in biopolyol.

## 5. Conclusions

This paper presented a synthesis of a new oil-based biopolyol for the production of RPU/PIR foams for thermal insulation applications. Evening primrose oil was used as a plant-based raw material, which had a high content of unsaturated fatty acids (unsaturated double bonds). Synthesis was carried out in a two-step method involving the epoxidation of double bonds and opening epoxy rings with diethylene glycol. The obtained biopolyol was characterized by a hydroxyl number of 182.41 mg KOH/g, an acid value of 0.84 mg KOH/g, and a water content of 0.2 wt.%. The new oil-based polyol was used as raw material for the synthesis of rigid polyurethane–polyisocyanurate foams from 0 to 0.4 mass equivalents of OH groups in a mixture with a commercial polyether polyol Rokopol RF-551. RPU/PIR foams were tested for use as thermal insulation materials. The conducted research has shown that the foams based on biopolyol had a slight lower apparent density, compressive strength, and brittleness. A slight improvement in the thermal insulation properties, closed cell contents, and aging resistance was also observed. The price of the finished product is also important. The production costs of this biopolyol are lower than the production costs of petrochemical polyol, which in the economic balance sheet gives a reduction in the price of final RPU/PIR foams. The use of evening primrose oil as an alternative raw material for the synthesis of green polyols offers great opportunities. The appropriate choice of composition for a reaction mixture enables obtaining various products, which may be used in various industries, such as for example in civil engineering, textile, furniture, footwear, or automotive industries. Besides, using plant-based polyols for the production of polyurethane materials is compatible with sustainable development principles and green chemistry philosophy. It enables the partial or complete replacement of petrochemical polyols.

## 6. Patents

The synthesis of biopolyol based on evening primrose oil was carried out on the basis of the patented method: Polish Patent Application number P.422888.

## Figures and Tables

**Table 1:** Amounts of the reactants in the two steps of synthesis of the biopolyol based on evening primrose oil (EPB) (g). EPO: evening primrose oil, EPEO: epoxidized evening primrose oil, AA: acetic acid, HP: hydrogen peroxide, SA: sulfuric acid, GDE: diethylene glycol.

	EPO	EPEO	AA	HP	SA	GDE	Efficiency (%)
1st step	1000.00	-	397.11	745.73	13.43	-	41.34
2nd step	-	1000.00	-	-	2.58	271.20	100.00

**Table 2:** Formulation of rigid polyurethane–polyisocyanurate (RPU/PIR) foams. DABCO: 1,4-diazabicyclo[2,2,2]octane.

Foam Symbol	Rokopol RF-551, (R) (g)	EPB (R) (g)	Tegostab 8460 (g)	33% DABCO (g)	33% Potassium Acetate (g)	Antiblaze TCMP (g)	Solkane HFC 365/227 (g)	Purocyn B (R) (g)
EPB2.0	1.0	0	4.59	2.70	6.75	45.90	32.40	3.0
	66.8	0						203.2
EPB2.1	0.9	0.1	4.74	2.79	6.97	47.48	33.44	3.0
	60.12	15.38						203.2
EPB2.2	0.8	0.2	4.89	2.87	7.19	48.86	34.49	3.0
	53.44	30.76						203.2
EPB2.3	0.7	0.3	5.03	2.96	7.40	50.34	35.53	3.0
	46.76	46.14						203.2
EPB2.4	0.6	0.4	5.18	3.05	7.62	51.82	36.58	3.0
	40.08	61.52						203.2

**Table 3:** Properties comparison of evening primrose oil, epoxidized oil, and biopolyol.

Parameter	EPO	EPEO	EPB
Smell	earthy	earthy	earthy
Iodine value (mol I2/100 g of fat)	0.658	0.386	0.386
Epoxy value (mol/100 g of fat)	0.000	0.253	0.000
Acid value (mg KOH/g)	8.720	-	0.840
Hydroxyl number (mg KOH/g)	8.720	-	182.410
Viscosity (mPa·s)	170	190	2400
Water content (wt.%)	0	0.6	<0.2
pH (-)	6.5	7.0	7.0
Molecular weight (g/mol)	820	917	1623

**Table 4:** Processing times of RPU/PIR foams with biopolyol.

Foam Symbol	Cream Time (s)	String Gel Time (s)	Tack Free Time (s)	Free Rise Time (s)
EPB2.0	8	21	23	34
EPB2.1	8	22	24	35
EPB2.2	9	23	25	35
EPB2.3	9	23	25	36
EPB2.4	9	23	25	37

**Table 5:** Flammability tests of RPU/PIR foams with biopolyol.

Foam Symbol	Combustion Residue (wt.%)	LOI vol % of O^2^)	Classification Based on PN-EN ISO 3582:2002
EPB2.0	92.77 ± 0.29	24.1 ± 0.1	self-extinguishing
EPB2.1	92.59 ± 0.34	24.1 ± 0.1	
EPB2.2	92.25 ± 0.32	24.0 ± 0.1	
EPB2.3	92.04 ± 0.27	24.0 ± 0.1	
EPB2.4	91.95 ± 0.36	23.9 ± 0.1	

**Table 6:** Results of SEM micrograph analysis.

Foam Symbol	Cell Size (μm)	Thickness of Cell Wall (μm)	Content of Cell per Area Unit (cell/mm^2^)
EPB2.0	258 ± 14	17 ± 2	12 ± 1
EPB2.4	269 ± 17	18 ± 2	11 ± 2

**Table 7:** Results of accelerated aging tests.

Foam Symbol	Change of Linear Dimension (%)	Change of Geometrical Volume (%)	Mass Loss (%)
EPB2.0	+0.96 ± 0.04	+2.75 ± 0.07	5.90 ± 0.12
EPB2.1	+0.92 ± 0.03	+2.61 ± 0.05	5.71 ± 0.06
EPB2.2	+0.91 ± 0.03	+2.53 ± 0.09	5.13 ± 0.10
EPB2.3	+0.87 ± 0.05	+2.46 ± 0.07	4.61 ± 0.09
EPB2.4	+0.85 ± 0.03	+2.39 ± 0.06	4.48 ± 0.15

## References

[1] Hojabri L., Kong X., Narine S.S. (2009). Fatty acid-derived diisocyanate and biobased polyurethane produced from vegetable oil: Synthesis, polymerization, and characterization. Biomacromolecules.

[2] Petrović Z.S., Guo A., Javni I., Cvetković I., Hong D.P. (2008). Polyurethane networks from polyols obtained by hydroformylation of soybean oil. Polym. Int..

[3] Baumann H., Bühler M., Fochem H. (1988). Natural Fats and Oils—Renewable Raw Materials for the Chemical Industry. Angew. Chem. Int..

[4] De Espinosa L.M., Ronda J.C., Galia M., Cadiz V. (2009). Plant oils: The perfect renewable resource for polymer science?. J. Polym. Sci. Part A Polym. Chem..

[5] Datta J., Glowinska E. (2014). Chemical modifications of natural oils and examples of their usage for polyurethane synthesis. J. Elastom. Plast..

[6] Mizera K., Ryszkowska J. (2018). Thermal properties of polyurethane elastomers from soybean oil-based polyol with a different isocyanate index. J. Elastom. Plast..

[7] Datta J., Głowińska E. (2014). Effect of hydroxylated soybean oil and bio-based propanediol on the structure and thermal properties of synthesized bio-polyurethanes. Ind. Crops Prod..

[8] Miao S., Zhang S., Su Z., Wang P. (2010). Vegetable-oil-based polymers as future polymeric biomaterials. J. Polym. Sci. Part A Polym. Chem..

[9] Behr A., Gomes J.P. (2010). The refinement of renewable resources: New important derivatives of fatty acids and glycerol. J. Lipid Sci. Technol..

[10] Palaskar D.V., Boyer A., Cloutet E., Le Meins J.-F., Gadenne B., Alfos C., Farcet C., Cramail H. (2018). Original diols from sunflower and ricin oils: Synthesis, characterization, and use as polyurethane building blocks. J. Polym. Sci. Part A Polym. Chem..

[11] Akram D., Hakami O., Sharmin E., Ahmad S. (2017). Castor and Linseed oil polyurethane/TEOS hybrids as protective coatings: A synergistic approach utilising plant oil polyols, a sustainable resource. Prog. Organ. Coat..

[12] Zhoua X., Sainab M.M., Oksman K. (2016). Semi-rigid biopolyurethane foams based on palm-oil polyol and reinforced with cellulose nanocrystals. Compos. Part A Appl. Sci. Manuf..

[13] Kadam H., Bandyopadhyay-Ghosh S., Malik N., Ghosh S.B. (2018). Bio-based engineered nanocomposite foam with enhanced mechanical and thermal barrier properties. J. Appl. Polym. Sci..

[14] Yu Z.L., Jiang C., Li W., Ma F.M., Wei S.C., Ruan M., Kong X., Han D.Y. (2018). Synthesis and Characterization of Polyurethanes from Oleic, Erucic and 10-Undecenoic Acids. Polym. Renew. Resour..

[15] Wang C., Yang L., Ni B., Wang L. (2009). Thermal and mechanical properties of cast polyurethane resin based on soybean oil. J. Appl. Polym. Sci..

[16] Mekewi M.A., Ramadan A.M., El Darse F.M., Abdel Rehim M.H., Mosa N.A., Ibrahim M.A. (2017). Preparation and characterization of polyurethane plasticizer for flexible packaging applications: Natural oils affirmed access. Egypt. J. Pet..

[17] Lubczak R., Szczęch D. (2018). Polyurethane foams with starch. J. Chem. Technol. Biotechnol..

[18] Lubczak R. (2016). Multifunctional oligoetherols and polyurethane foams with carbazole ring. Pol. J. Chem. Technol..

[19] Chmiel E., Lubczak J. (2018). Synthesis of oligoetherols from mixtures of melamine and boric acid and polyurethane foams formed from these oligoetherols. Polym. Bull..

[20] Kurańska M., Prociak A. (2014). Environmentally friendly polyurethane-polyisocyanurate foams for applications in the construction industry. Czasopismo Techniczne Budownictwo.

[21] Prociak A., Rokicki G., Ryszkowska J. (2014). Polyurethane Materials.

[22] Kirpluksa M., Kalnbundea D., Benesb H., Cabulis U. (2018). Natural oil based highly functional polyols as feedstock for rigid polyurethane foam thermal insulation. Ind. Crops Prod..

[23] Chen M.-J., Wang X., Tao M.-C., Liu X.-Y., Liu Z.-G., Zhang Y., Zhao C.-S., Wang J.-S. (2018). Full substitution of petroleum-based polyols by phosphorus containing soy-based polyols for fabricating highly flame-retardant polyisocyanurate foams. Polym. Degrad. Stab..

[24] Paciorek-Sadowska J., Borowicz M., Czupryński B., Tomaszewska E., Liszkowska J. (2018). Oenothera biennis seed oil as an alternative raw material for production of bio-polyol for rigid polyurethane-polyisocyanurate foams. Ind. Crops Prod..

[25] Abdel Hakim A.A., Nassar M., Emam A., Sultan M. (2011). Preparation and characterization of rigid polyurethane foam prepared from sugar-cane bagasse polyol. Mater. Chem. Phys..

[26] Ionescu M. (2016). Chemistry and Technology of Polyols for Polyurethanes.

[27] Paciorek-Sadowska J., Borowicz M., Czupryński B., Tomaszewska E., Liszkowska J. (2018). New bio-polyol based on white mustard seed oil for rigid PUR-PIR foams. Pol. J. Chem. Technol..

[28] Paciorek-Sadowska J., Borowicz M., Czupryński B., Liszkowska J. (2017). The Method of Obtaining a Polyol Raw Material for the Synthesis of Rigid Polyurethane-Polyisocyanurate Foams. Polish Patent.

[29] ASTM International (2016). Standard Practice for Polyurethane Raw Materials: Polyurethane Foam Cup Test.

[30] Dworakowska S., Bogdał D., Prociak A. (2012). Microwave-Assisted Synthesis of Polyols from Rapeseed Oil and Properties of Flexible Polyurethane Foams. Polymers.

[31] Septevani A.A., Evans D.A.C., Chaleat C., Martin D.J., Annamalai P.K. (2015). A systematic study substituting polyether polyol with palm kernel oil based polyester polyol in rigid polyurethane foam. Ind. Crops Prod..

[32] Zhan G., Zhao L., Hu S., Gan W., Yu Y., Tang X. (2008). A novel biobased resin epoxidized Soybean oil modified cyanate ester. Polym. Eng. Sci..

[33] Zhang L., Huang M., Yu R., Huang J., Dong X., Zhang R., Zhu J. (2014). Bio-based shape memory polyurethanes (Bio-SMPUs) with short side chains in the soft segment. J. Mater. Chem. A.

[34] Sripathy M., Sharma K.V. (2013). Flammability and Moisture absorption test of rigid polyurethane foam. Int. J. Sci. Eng. Res..

[35] Paciorek-Sadowska J., Borowicz M., Czupryński B., Liszkowska J. (2016). Use of volcanic tuff for production of rigid polyurethane-polyisocyanurate foams. Przem. Chem..

[36] Zhang H., Fang W., Li Y., Tao W. (2016). Experimental study of the thermal conductivity of polyurethane foams. J. Appl. Therm. Eng..

